# Synthetic political actors: an exploration into emerging AI-driven candidates and parties

**DOI:** 10.12688/openreseurope.23686.1

**Published:** 2026-04-30

**Authors:** Michal Malý, Giulia Sandri, Oscar Barberà

**Affiliations:** 1 Charles University Institute of Political Studies, Prague, Prague, Czech Republic; 2 Universite Libre de Bruxelles, Brussels, Brussels, Belgium; 3 Universitat de Valencia Facultat de Dret, Valencia, Valencian Community, Spain

**Keywords:** synthetic political actors, artificial intelligence, political representation, political parties, synthetic politics, digital politics, algorithmic democracy

## Abstract

**Background:**

Artificial intelligence is becoming more visible in democratic politics not only as a campaign tool or communication aid, but also as a publicly presented political persona. In several democratic contexts, AI-based candidates and parties have emerged that invite citizens to interact with synthetic interfaces as if they were political representatives, leaders, or candidates in their own right. This article conceptualises these initiatives as synthetic political actors and examines them as an emerging phenomenon in democratic politics.

**Methods:**

The article draws on scholarship on party politics and digital politics to develop a six-dimensional analytical framework covering political goals, political agency, normative assumptions, entrepreneurship, organisational structure, and participation. It examines ten synthetic political candidates and parties across eight national contexts through qualitative document analysis and theoretically informed thematic analysis.

**Results:**

Synthetic political actors do not form a coherent ideological or organisational family. They differ in how they distribute authority between humans and AI systems, how they justify the political role of AI, and how they organise participation and representation. Some pursue institutional electoral ambitions, while others remain primarily symbolic, demonstrative, or experimental. In most cases, synthetic politics is marked by limited organisational development, interface-centred participation, and a shift away from party-based linkage towards algorithmically mediated representation.

**Conclusions:**

Synthetic political actors are best understood as a diverse and still developing set of initiatives rather than a uniform category of political actor. The article offers a comparative framework for analysing this phenomenon and shows how these cases complicate established understandings of democratic delegation, representation, and linkage. Their emergence also underlines the need for further research on their institutional trajectories, democratic implications, and regulatory treatment.

## Introduction

Artificial intelligence (AI) has become an increasingly visible element of contemporary politics. AI systems are increasingly being adopted in electoral campaigning and political communication ( Foos 2024, Jungherr et al. 2026, Novelli et al. 2024, Scherer et al. 2026). That said, AI systems are also publicly presented in ways that extend beyond backstage support functions. Media reports and political initiatives across different countries refer to AI candidates, AI representatives, or AI-led political projects, in which citizens are invited to interact directly with AI in political contexts ( Schneier and Sanders 2025). These developments indicate a shift in how AI is positioned within political processes, moving from an auxiliary technology toward a more publicly recognisable role in political representation.

The rapid proliferation of this phenomenon is closely linked to the widespread adoption of large language models (LLMs) and conversational AI systems. The mass diffusion of tools such as ChatGPT after 2022, together with the normalisation of generative AI in public and political communication, has accelerated the emergence of new AI-driven political initiatives ( Chatterji et al. 2025). Whereas earlier experiments with AI-driven political actors were often symbolic, artistic, or protest-oriented ( García-Marzá and Calvo 2024), recent years have brought a diversification of formats and ambitions, including attempts to contest elections, facilitate proxy representation, and mobilise voters ( Vuković 2025). Yet the existing literature on AI and politics has largely approached AI as a tool embedded in established political processes rather than as a political actor in itself. Most studies examine its role in electoral campaigning ( Foos 2024, Núñez et al. 2025), political communication ( Hackenburg and Margetts 2024), and voter persuasion ( Lin et al. 2025), while campaign-focused scholarship has also analysed the spread of AI-generated synthetic content, including deepfakes ( Altman 2026, Bontridder and Poullet 2021, Vaccari and Chadwick 2020). A smaller body of research explores internal party uses, such as decision-support systems or experiments with intra-party participation ( Novelli et al. 2024; Yardımcı-Geyikçi et al. 2026), alongside the deployment of conversational agents for campaigning and voter engagement ( Ben-David and Carmi 2025, Kim and Lee 2023, Tosi et al. 2026). Even where AI agents adopt personalised or human-like personae, as in the case of Likud’s Bibi bot ( Ben-David and Carmi 2025), they remain conceptualised primarily as auxiliary instruments of human-led parties and candidates.

We describe these initiatives as *synthetic* because political agency is performed through a persona generated at the intersection of algorithmic systems and human oversight. The political figure encountered by citizens is neither a traditional human representative nor a fully autonomous machine, but a hybrid construction produced through interaction with an AI interface built by someone. Similar to the use of synthetic populations in social science and marketing research, in which (some or all) participants are modelled agents, synthetic political actors’ emergence raises issues related to known LLM limitations, such as standardization biases, sycophantic responses, and deceptive behaviours, but also to erosion of political agency and weakened democratic accountability ( Argyle et al. 2023, Hradec et al. 2023, Petit and Pachot 2025). We also argue that such emerging synthetic political actors represent a distinct new phenomenon within contemporary politics that existing approaches to AI in politics are still struggling to capture, as it generates significant consequences for political representation and decision-making delegation ( Sandri 2026).

So far, cutting-edge research from political communication and philosophy has focused on a limited number of early examples of synthetic political actors, often examining their rhetorical strategies, normative claims, or symbolic significance ( García-Marzá and Calvo 2024, Nasi 2025, Staunæs and Herrie 2024, Vuković 2024 and 2025). Overall, comparative empirical analysis remains scarce, particularly from an organisational perspective. The aim of this article is to addresses this gap by engaging into a more in-depth theoretical exploration and empirical assessment of the nature and key features of, ten synthetic political candidates and parties across multiple national contexts. Drawing extensively from the party politics literature, the paper examines the main goals of this emerging group of political actors, how they articulate political representation, how political agency is distributed between AI systems and human actors, or how they conceive internal organisation and participation.

The rest of the article proceeds as follows. The next section develops a theoretical framework by situating synthetic political actors within the broader field of digital politics and classical debates on political agency, organisation and representation. The subsequent section outlines the data, methods, and case selection strategy. This is followed by the empirical analysis, which examines how synthetic political candidates and parties engage with electoral politics, articulate political representation, and configure the relationship between AI and human agency. The article concludes by discussing the implications of these findings and identifying avenues for future research.

## Assessing the nature and main features of emerging synthetic political actors

Political parties are classically understood as institutionalized mediators between civil society and the state, performing a cluster of interrelated roles: soliciting and articulating public policy priorities, socializing and educating voters in the functioning of the political system, balancing opposing demands and converting them into general policies, and activating and mobilizing citizens to participate in political decision ( Katz and Crotty 2006). They also recruit candidates, structure electoral competition, and organize legislative coordination once in office ( Sartori 1976).

AI systems generating and disseminating policy-consistent messaging through AI-based synthetic interfaces, segmenting and targeting constituencies, simulating responsiveness to public demands via AI-personae, or coordinating political narratives at scale, indeed replicate the functional profile of political parties in ways that existing paradigms for studying political technology do not adequately capture. It is precisely this functional equivalence, not any claim to consciousness or formal legal standing, that warrants classifying such systems as synthetic political actors. In this regard, the literature has so far identified as synthetic political actors all those political initiatives organised around AI-based synthetic interfaces that are presented as recognisable political personae and serve as central points of political engagement constitute what the literature ( Staunæs and Herrie 2024, Vuković 2025). The designation of synthetic candidates and personas as political actors is therefore functional, not merely metaphorical. Synthetic interfaces indeed constitute the core political actor around which the entire project is organised and enables political addressability: citizens can ask questions, express preferences, and receive political responses. They also serve as the primary medium through which political representation, participation, and communication are enacted: they articulate policy positions, respond to political demands, or mediate political participation ( Staunæs 2026). In doing so, they reposition AI from a supportive instrument to a focal site of political agency, blurring the boundary between technological tool and political actor. This broader shift also resonates with recent work on AI and democracy that stresses the sociotechnical character of AI’s democratic effects. Nasi (2025) argues that AI may exacerbate, alleviate, or even help transform aspects of the democratic crisis depending on the context in which it is embedded. This perspective is useful here because it encourages us to analyse synthetic political actors not as purely technical artefacts, but in relation to the political and organisational settings in which they are constructed and deployed.

So far, the literature has mostly discussed political agency and algorithmic representation as two of the main features of synthetic political actors ( García-Marzá and Calvo 2024, Nasi 2025, Staunæs and Herrie 2024, Vuković 2024 and 2025).

The first feature examines the configuration of political agency between humans and AI systems and assesses how each synthetic political actor publicly constructs authority. In this regard, Vuković’s (2025) built a typology differentiating autonomous chatbot actors, hybrid human–AI candidates, and digital replicas or avatars, depending on how political agency is distributed between human actors and AI systems. Vuković’s also highlighted the distinction between *political chatbots*—campaign or communication tools embedded in human-led organisations—and *chatbot politicians*, which are publicly presented as candidates or representatives themselves. This typology has been complemented by an augmentation–replacement continuum ( Alfieri et al. 2026), which captures whether AI is framed as supporting human political judgement or whether humans’ function primarily as proxies committed to implementing AI-generated outputs. It is important to point out that the central question is not whether AI is autonomous, but how authority (agency) is distributed, justified, and operationalised in practice. Rather than a claim about machine autonomy, this dimension deals with the internal configuration of political agency and authority. Following this logic, Staunæs and Herrie (2024) paper analysed to what extent the Danish *Synthetic Party* was staged as a political actor. Focusing on the party’s chatbot leader, Leader Lars, as the central representative and decision-making interface, they described a model of algorithmic or synthetic representation in which political programmes are generated through AI-mediated aggregation of citizen input. The Synthetic Party indeed seems a good example of how the AI interface has the main political agency, while humans operate in the backstage as organisers and legal intermediaries ( Vuković 2025).

The second dimension is closely interrelated with the former and discusses the normative assumptions embedded in design choices, including how authority is legitimised and how representation is operationalised ( Coeckelbergh 2020). Rather than approaching synthetic political actors through ideology in the classical sense of a coherent set of ideas guiding organised political action ( Heywood 2021), it is more useful to focus on the narrower normative claims they advance about the role of AI in politics, especially since the existing literature has so far not identified a coherent ideology underlying these actors. Critiques of representative democracy converge here with techno-optimist claims about efficiency, neutrality, or algorithmic rationality. In this sense, García-Marzá and Calvo’s (2024, 51–57) seminal study approached synthetic candidates and parties through the lens of algorithmic democracy. They conceptualised synthetic political actors as attempts to delegate representation and decision-making to algorithmic systems, emphasising both their democratic promises—such as responsiveness, objectivity, and algorithmic neutrality—and their risks, particularly regarding transparency, responsibility, and the erosion of human judgement. Their analysis treated synthetic political actors primarily as instances of algorithmic replacement, where AI systems are expected to substitute, at least partially, human political agency. Normatively, Vuković (2025) situated these developments within debates on technopopulism, highlighting how claims of neutrality, rationality, and incorruptibility have been mobilised to legitimise the delegation of political agency to AI, while questions of accountability and control remain opaque. At the same time, such claims may also reproduce existing illiberal discourses under the guise of efficiency and objectivity.

Beyond political agency and normative assumptions about politics or representation, synthetic political actors are forms of collective action that resonate with previous discussions of new politics. Earlier waves of innovation in politics indeed emphasised experimentation with organisational forms, leadership structures, modes of political mobilisation and ideological positioning beyond established party cleavages ( Bolleyer 2013, Panebianco 1988, Poguntke 1987, Rahat 2025). In this regard, the most obvious precedent is the wave of new digital parties that emerged in many European democracies by the 2010s ( Barberà et al. 2021, Gerbaudo 2018). However, this organisational approach has been somewhat neglected when dealing with synthetic political actors. That is why the rest of this section we are going to synthetically discuss four additional main features based on the party politics literature (see Table 1).

a) *Political goals.* Drawing on party goals theory ( Harmel and Janda 1994, Strøm 1990), this dimension examines what kind of political aims synthetic political actors pursue. It asks whether these initiatives articulate office-seeking, vote-seeking, or policy-seeking ambitions, but also whether they are oriented towards broader symbolic or interventionist goals. While some seek to compete electorally and gain office through conventional institutional channels, others function primarily as agenda-setting projects, symbolic candidacies, forms of critique, or experiments in alternative modes of representation.
b) *Political entrepreneurship.* As previously stated, the emergence of synthetic political actors has more to do with political agency and authority distribution than with machine autonomy or human replacement. That is why the human dimension of synthetic politics cannot be disregarded. Such analysis should consider who initiates and designs these projects since political values are deeply embedded in political form and the technological architecture underpinning it. The key question is who stands behind these initiatives and how their backgrounds shape organisational structure, authority distribution, and models of political mediation ( Buyl et al. 2026). This is something that the literature on party politics has long established, mostly pointing out the differences between individual and collective political entrepreneurship ( Bolleyer 2013, Duverger 1954; Panebianco 1988). Research on digital parties has also shown that technologically embedded milieus and platform developers have often shaped organisational innovation and participation models ( Burkart 2014, Deseriis and Vittori 2019).
c) The *organisational structure* provides an analytical dimension linking synthetic actors to established party theory. Like in the previous dimension, the main rationale here considers that underpinning successful (long term) collective action there is always some form of organisational structure. In this regard, traditional scholarship has emphasised organisational depth, internal hierarchies or faces, and embeddedness in civil society ( Duverger 1954, Janda 1980, Katz and Mair 1995, Kitschelt 2006, Panebianco 1988). More recent research on digital politics has pointed out the interconnections between party structure and organisation and their digital platforms ( Lioy et al. 2019, Lisi 2025, Sandri et al. 2025). In this regard synthetic political actors seem to be structured primarily as interface-based projects with minimal organisational infrastructure, but they may develop as well broader organisational arrangements including affiliated teams, offline organisational elements, etc. The party politics literature has also highlighted the relevance of the structure-leadership dilemma in shaping the future evolution of political parties ( Bolleyer 2013).
d) *Internal participation.* This dimension considers the form of participation offered to citizens, members or affiliates. Internal participation has also been one of the key features discussed by the party politics literature. Duverger’s (1954) seminal work (the so call concentric circles model) connected participation with organisation and party types. This logic has ever since permeated the subsequent literature on this topic. The impacts of the digital transformation on the internal participation in political parties have long been discussed by the literature ( Gibson and Ward 2009, Norris 2001). One of the most influential models reflecting emerging modes of offline/online participation is the multi-speed model developed by Scarrow (2015). That said, even in the previous wave of digital parties, participation was articulated in more or less conventional forms of membership and affiliation. Conversely, synthetic political actors organise interaction (and participation) through entirely alternative approaches. Through AI-mediated conversational interfaces ( Taylor and Richey 2026), citizens may engage in dialogue, submit preferences, or provide inputs that are aggregated and processed algorithmically. In this configuration, participation operates through data extraction and computational mediation, positioning citizens as inputs within a system of algorithmic decision-making ( Zuboff 2019) and representation ( Rouvroy 2020). In some cases, citizens also serve as procedural or legal proxies for AI-based candidacies. This dimensions indeed reflects some of the most striking changes with past models of party organisation.

**Table 1.** Main analytical dimensions for the study of emerging synthetic political actors.

Dimension	Key features
**Political goals**	Analyses statements both in terms of functions and political strategies (office, votes, policies).
**Political Agency**	Assesses how authority is distributed between human actors and AI systems and how decision-making is justified and operationalised.
**Normative assumptions**	Examines how representation and democracy is conceptualised, and what (anti) representative ideas are embedded and underpin the project.
**Entrepreneurship**	Describes the background and positionality of individuals or collectives initiating the project. Evaluates how social and professional milieus shape authority distribution, institutional design, and models of political mediation.
**Organisational structure**	Highlights the presence of organisational structures beyond the AI interface: affiliated teams, social media ecosystems, offline branches, and organisational depth, etc.
**Participation**	Assesses modes of citizen engagement through AI interfaces. Examines whether participation functions as deliberation, preference input, data contribution, or proxy representation, and what political weight such input carries.

Source: Author’s own elaboration.

Taken together, these six dimensions enable a structured comparison of the nature and main features of several emerging synthetic political actors without presupposing their coherence, durability, or institutional trajectory. They also allow to move beyond the dominant “AI as tool” perspective and to examine how synthetic agency is positioned, justified, and institutionalised within democratic electoral politics.

## Case selection, data and methods

The emergence of synthetic political actors can be traced back to 2017, when the Semantic Analysis Machine (SAM) was launched in New Zealand as a conversational political project seeking electoral relevance. Subsequent initiatives followed in different national contexts, including Alisa, introduced during the 2018 Russian presidential elections ( García-Marzá and Calvo 2024: 44–46). While early cases remained relatively isolated and often experimental, the diffusion of commercial LLMs after 2022, particularly systems developed by companies such as OpenAI and Anthropic ( Chatterji et al. 2025), coincided with a marked increase in the number, visibility, and organisational sophistication of AI-driven political actors. The post-2022 period thus represents an imaginary second wave characterised by broader public uptake and stronger claims to political agency.

Synthetic political actors have emerged across diverse institutional environments. Some have been framed as electoral candidates or party leaders, while others have appeared in advisory or institutional roles. A prominent example is Diella in Albania, launched in 2025 and presented as the world’s first AI minister ( Tika 2025), alongside initiatives such as ION in Romania, introduced in 2023 as a governmental AI adviser ( Preussen 2023). Additional projects include AI Mark, unveiled in 2025 as the first AI MP and described as a synthetic copy of Labour Party MP Mark Sewards in the United Kingdom ( Moss and Vincent 2025), as well as the Belarusian synthetic dissident Yas Gaspadar, launched in 2024 ( Ticălău 2025 **)**. These cases illustrate the range of contexts in which AI personas have been embedded in the different political settings. However, not all these initiatives are analytically comparable. For the purposes of this article, we focus exclusively on cases that meet three criteria: (1) the initiative presents an AI-based persona as a political candidate or party actor (e.g. party leader); (2) it has participated in, or explicitly declared an intention to participate in, electoral competition; and (3) it operates within a democratic political system. Cases in which synthetic personas were installed directly within existing institutions, functioned solely as advisory or symbolic interventions without electoral ambition, or have since been fully dissolved without ongoing political activity are excluded.

Applying these criteria results in a sample of ten synthetic political candidates and parties. Seven of these cases were identified through participation in the Synthetic Summit 2025, held in Denmark, which constituted the first international gathering of synthetic political actors ( Staunæs 2026). The summit brought together eight initiatives; one case (Simiyya) was excluded from our analysis because it did not seek electoral participation. The remaining seven cases form the core of our sample: Wiktoria Cukt 2.0 (Poland); Parker Politics in New Zealand, building on the earlier SAM project; Olof Palme (Sweden); Lex AI (Brazil); Koneälypuolue (Finland); AI Mayor (Japan); and Leader Lars (Denmark). To broaden the scope beyond summit participants, we added three further cases that are publicly documented and discussed in the emerging literature ( Schneier and Sanders 2025), namely AI Steve (United Kingdom), VIC (Virtual Integrated Citizen) in the United States, and AI Anno (Japan). The full set of cases, including country, year of launch, organisational affiliation and creator is presented in Table 2.

**Table 2.** Overview of selected synthetic candidates and parties.

Political Actor	Affiliation	Entrepreneur(s)	Country	Year founded
**AI Anno**	Team Mirai	Takahiro Anno	Japan	2024
**AI Mayor**	Japanese AI Party	Michihito Matsuda	Japan	2019
**AI Steve**	Smarter UK	Steve Endacott	UK	2024
**Leader Lars**	Synthetic Party	Asker Bryld Staunæs	Denmark	2022
**Lex AI**	None	Pedro Markun	Brazil	2022
**Koneälypuolue**	Finnish AI Party	Samee Haapa	Finland	2018
**Olof Palme**	Swedish AI Party	Emma Bexell	Sweden	2024
**Parker Politics** *	None	Nick Gerritsen	New Zealand	2017
**VIC**	None	Victor Miller	USA	2024
**Wiktoria Cukt 2.0** **	None	The collective C.U.K.T.	Poland	2024

Source: Syntheticism.org (2025) and Author’s own elaboration.

^*^ Parker’s previous version was SAM

^**^ Wiktoria Cukt was first launched in 2001 as an art project and revived in 2024 as a Synthetic Candidate

This study adopts a qualitative, exploratory research design aimed at systematically examining synthetic political candidates and parties as an emerging political phenomenon. Given their recent development and limited institutionalisation, the objective is analytical clarification and structured comparison rather than statistical representativeness or causal inference. The design follows established case-oriented research principles that prioritise theoretically relevant and information-rich cases when studying novel or weakly consolidated phenomena ( George and Bennett 2005, Gerring 2007).

Empirically, the study is based on qualitative document analysis. Data were collected from AI interfaces, official project websites, manifestos, public statements, media reports, and secondary academic sources. Where platforms were no longer accessible, archived webpages and documented outputs were used. The use of multiple source types enables basic triangulation and strengthens the reliability of case reconstruction. To enhance transparency and reproducibility, the study is accompanied by an author-compiled dataset covering all ten synthetic political actors included in the analysis ( Malý 2026).

Thematic analysis ( Braun and Clarke 2006) was performed on the selected documents to assess the main features from the six dimensions outlined in the previous section. The analysis proceeds as a structured comparative mapping guided by the abovementioned analytical dimensions. Each case was examined in relation to its declared political goals, the distribution of human and AI agency, its model of representation and participation, organisational structure, and creator background. The aim is not to reconstruct technical architectures in detail, but to identify recurring patterns in how synthetic political actors position AI as a locus of political authority within democratic electoral contexts.

## Synthetic candidates and parties: an exploratory comparative assessment

The empirical analysis proceeds as a structured comparative assessment of synthetic political actors across the six analytical dimensions developed in the theoretical section: political goals, political agency, normative assumptions, entrepreneurship, organisational structure, and participation. Rather than presenting each case separately, the analysis is organised dimension by dimension in order to identify recurring patterns, internal variation, and broader tendencies across the phenomenon as a whole. The focus is therefore not on the individual cases per se, but on what they collectively reveal about the emerging logic of synthetic political actors in democratic politics.

### Political goals

Drawing on party goals theory, the political aims of synthetic political actors can be interpreted in terms of office, votes, and policy influence, but only if those categories are used flexibly. The cases do not share a single strategic orientation. Instead, they spread across a continuum ranging from attempts at institutional electoral entry to symbolic and experimental interventions that use the language or staging of politics without treating office acquisition as the primary destination.

At one end stand *institutional-electoral* projects. These are cases in which elections function as a serious route into institutional politics and, in some instances, as a pathway toward more durable party formation. AI Mayor is the example of repeated electoral ambition at the local and regional levels. Unlike most cases in the dataset, it has a party structure, headquarters, and a longer electoral trajectory. AI Anno moves even further in this direction. What began as an AI-enhanced gubernatorial campaign in Tokyo developed into Team Mirai, which first entered the House of Councillors in 2025 and then gained parliamentary representation in the House of Representatives in 2026. Here, AI was one of the tools that helped the new party appeal to a broad electorate.

A second group is best described as *electoral-demonstrative.* In this group synthetic actors contest elections, but they also serve as public tests of AI-mediated representation, governance, or responsiveness. AI Steve, Lex AI, and VIC all entered actual electoral contests but remained electorally marginal. Their campaigns were therefore as many demonstrations of possibility as credible bids for office. This is especially important in VIC and, in a different form, AI Mayor, where the human candidate functions as a legal and institutional proxy for a synthetic political actor. In such cases, the goal is not simply to elect a human politician who uses AI tools, but to bring an AI-centred project into the representative arena through a person who can legally stand on the ballot. The Leader Lars and Olof Palme also belong close to this zone, though in a more overtly agenda-setting and performative form.

At the other end of the continuum are *symbolic-experimental* projects. Wiktoria Cukt 2.0 uses candidacy as a critical intervention into representative democracy rather than as a serious effort to win office. Koneälypuolue similarly remains closer to political experimentation and performative imagination than to conventional electoral competition. Parker Politics is less overtly artistic, but it too is better understood as a project of mediated democratic engagement and policy responsiveness than as a verified electoral actor. These cases still pursue political goals, but they do so primarily through visibility, critique, and experimentation rather than through institutional office-seeking.

Taken together, the evidence suggests that synthetic political actors do not share one unified electoral logic. Some seek to institutionalise themselves through repeated candidacies and party-building; others use elections as a demonstrative arena in which AI can be publicly tested; and still others remain primarily symbolic or experimental interventions into the meaning of representation and leadership. What unites them is not a common strategy of office acquisition, but a shared effort to stretch the boundary between electoral competition, public performance, and technological experimentation. The comparative clustering of these political goals and the corresponding electoral outcomes is summarised in Table 3.

**Table 3.** Political goals of synthetic political actors.

Cluster	Case	Political goals	Result
**Electoral-institutional **	AI Mayor	Seeks to institutionalise AI-centred politics through repeated electoral entry, party organisation, and the use of human candidates as legal and electoral vehicles for an AI-centred political project.	Failed in Tama City Assembly (275 votes, 31/32), Tama mayoral election (516 votes, 3/3), and Tokyo gubernatorial election (2,761 votes, 17/56), but the party’s history also includes a successful endorsed local candidacy in Kosaka in 2024
	AI Anno	Began as a serious digital-democracy candidacy and evolved into an institutional party-building project with national parliamentary representation.	154,638 votes, 5/56 in Tokyo (2024); Team Mirai then won 1 Upper House seat in 2025 with 1,517,890 PR votes and 11 House of Representatives seats in 2026 with 3,813,749 PR votes
**Electoral-demonstrative **	AI Steve	Uses a real electoral candidacy to demonstrate AI-mediated responsiveness and policy co-creation, while also testing whether a chatbot-centred campaign can be politically credible	179 votes, 8/8, not elected
	VIC	A genuine mayoral bid, but also a public experiment in AI-led governance. The human candidate functions as a legal proxy through whom the AI actor can enter the electoral arena.	327 votes, 4/6, did not advance
	Lex AI	A real local candidacy designed not only to seek office, but to demonstrate hybrid human–AI lawmaking, transparency, and civic-tech governance.	627 votes; not elected; listed as suplente
	Leader Lars	Uses the electoral arena as a demonstrative and agenda-setting platform for algorithmic politics and the representation of non-voters.	Did not reach the ballot because it failed to secure enough signatures
	Olof Palme	Combines electoral ambition with performative political intervention; elections matter, but they also serve as a stage for reimagining synthetic leadership and democratic dialogue.	No electoral result yet
**Symbolic-experimental **	Wiktoria Cukt 2.0	Uses the form of candidacy as a critical intervention into representative democracy rather than as a credible route to office.	Symbolic, unofficial candidacy
	Koneälypuolue	Oriented primarily toward political experimentation and the imaginative testing of AI-led politics rather than immediate office acquisition.	No confirmed election participation
	Parker Politics	Focuses on democratic engagement, voice amplification, and mediated responsiveness rather than verified office-seeking.	No confirmed election participation

Source: AI Mayor (2026), Bexell (2025), Sanders and Schneier (2026), Schneier and Sanders (2025), Syntheticism.org (2025), Vuković (2025)

### Political agency

Synthetic political actors can be clustered in three different groups according on their conception of political agency. The first most distinctive one consists of those actors in which AI is publicly staged as a new and main political actor rather than as a support tool for an existing human politician. This category includes AI Mayor, Leader Lars, Koneälypuolue, Olof Palme, Parker Politics, and Wiktoria Cukt 2.0. In these cases, the AI interface constitutes the main point of political interaction and public visibility. Citizens are invited to engage with the synthetic persona as if it were the candidate, leader, or central representative itself. These projects thus come closest to constructing genuinely new political actors ( Rahat 2025), even if human designers remain indispensable in the background.

A second category is formed by digital replicas, where AI does not create a fully distinct political subject but instead extends the public persona of an already existing leader. This applies to AI Anno and AI Steve, both of which function as digital replications of identifiable human politicians. Here, AI primarily augments leadership and can be understood as a form of leader-augmented personalisation. Rather than displacing the human political entrepreneur, the synthetic layer strengthens their accessibility, scalability, and responsiveness. Political agency thus remains anchored in the human leader, while AI serves as an extension of their representative presence.

Finally, Lex AI and VIC represent hybrid human–AI actors, occupying an intermediate position between synthetic autonomy and leader augmentation. In these cases, political agency is shared more explicitly between the AI system and human actors. AI is neither merely a replica of a leader nor fully constructed as an independent political subject. Instead, these initiatives combine human oversight, strategic control, and political entrepreneurship with a visible AI-mediated representative role. These three configurations of political agency are summarised in Table 4.

**Table 4.** Level of political agency of synthetic political actors.

Cluster	Autonomous	Digital replica	Hybrid human-AI
**Cases**	AI Mayor, Leader Lars, Koneälypuolue, Olof Palme, Parker Politics, Wiktoria Cukt	AI Anno, AI Steve	Lex AI, VIC

Source: Author’s own elaboration based on Vuković (2025)

### Normative assumptions

The ten cases do not point to a single normative model of synthetic politics. They fall more convincingly into three clusters that differ in how they justify the political role of AI. The first cluster consists of projects that present AI as a means of broadening representation and deepening responsiveness. In these cases, AI is used to expand the capacity of political actors to listen, process input, and maintain ongoing contact with citizens. Parker Politics is framed around democratic re-engagement and the amplification of voices that are usually weakly heard in conventional politics ( Salole 2024). AI Steve follows a similar logic, but in a more electoral form. Its central claim is that representation can be improved when a candidate is continuously tied to constituent feedback and policy formation is opened to structured public input ( SmarterUK 2024). Takahiro Anno’s campaign in Japan fits this pattern as well, since it relied on AI-enabled broad listening and large-scale collection of voter concerns rather than on the idea that AI should replace human political judgement altogether ( Sanders and Schneier 2026). Leader Lars partly belongs here too, although in a more confrontational form, because the Synthetic Party is built around the claim that existing representative institutions leave a significant part of the electorate, especially non-voters, without meaningful representation ( Staunæs and Herrie 2024). In this first cluster, AI functions above all as an instrument for widening access, strengthening responsiveness, and reworking the link between representation and participation.

A second cluster is more clearly technocratic. Here the legitimacy of AI rests less on inclusion and more on claims of objectivity, transparency, informational capacity, and better decision-making. Lex AI was presented as a hybrid candidacy built around data-driven governance, oversight of public spending, and AI-assisted policy design ( Riquelme 2024). VIC was justified in similar terms, with the argument that a chatbot-guided administration could make more rational and legally coherent decisions than ordinary politicians ( Betts 2024). The Japanese AI Mayor project also belongs in this group, despite its participatory language, because its public rationale repeatedly stresses transparent, evidence-based, and more balanced municipal governance ( Oppo 2025). These cases are distinct from the first cluster because they do not primarily seek to scale democratic voice; instead, they seek to correct perceived weaknesses of human government through better information processing, reduced corruption, and more consistent judgement. What holds them together is a shared assumption that AI may improve democratic legitimacy when it is treated as a superior instrument of governance rather than only a better channel of communication.

The third cluster includes cases in which AI is used to question the human and institutional foundations of politics more directly. Wiktoria Cukt 2.0 is the clearest example, as it revives an anti-parliamentary tradition and links AI politics with direct electronic democracy and the marginalisation of human intermediaries ( Wyrzykowski 2024). The Finnish AI Party emerged from an art-political environment that treated AI not simply as an administrative tool but as a challenge to the assumption that humans should remain the exclusive agents of governance ( The Center for Everything 2017). The Olof Palme project belongs here as well, since it experiments with non-human leadership and with the possibility that ideological direction can be simulated, iterated, and publicly reprogrammed through synthetic political figures ( Bexell 2025). These projects are harder to place within ordinary electoral categories, but that is precisely the point: their normative ambition lies in exposing the limits of conventional representation and testing post-human alternatives. Read together, the three clusters suggest that synthetic political actors are not a coherent ideological family. They form a broader field in which AI is used, respectively, to enhance representation, correct governance, or unsettle the human premises of politics itself. The three clusters of normative assumptions identified across the cases are summarised in Table 5.

**Table 5.** Three clusters’taxonomy of normative assumptions.

Cluster	Case	Normative assumption
Participatory-representational	AI Anno	AI-enhanced broad listening and co-creation
	AI Steve	Continuous representation anchored in constituent feedback
	Leader Lars	Representation of non-voters through algorithmic politics
	Parker Politics	Re-engagement of citizens through AI-mediated responsiveness
Technocratic-governance	Lex AI	Transparent and data-driven civic governance
	VIC	Rational and less corrupt governance through AI assistance
	AI Mayor	Participatory and evidence-based local governance
	Wiktoria Cukt 2.0	Direct electronic democracy beyond human intermediaries
Art-political/post-human	Finnish AI Party	AI as an alternative to failed human governance
	Olof Palme	Synthetic ideological leadership beyond human limitations

Source: Author‘s own elaboration

### Entrepreneurship

The entrepreneurship dimension is best approached here through the orientation of founders rather than through formal origin types from the party politics literature ( Duverger 1954, Panebianco 1988). Research on political recruitment, candidate selection, and party organisation has long treated the social and professional backgrounds of political actors as analytically relevant ( Borchert and Zeiss 2003, Carnes and Lupu 2023). In the case of synthetic political actors, this perspective is especially useful because these projects do not emerge as neutral technological artefacts. They are initiated and curated by identifiable individuals or collectives whose professional experience, creative orientation, and technical expertise leave visible traces in the political form of the project itself ( Bolleyer 2013).

The ten cases in the dataset suggest three broad clusters. A first cluster is artistic. It includes projects such as Leader Lars, Koneälypuolue, Olof Palme, and Wiktoria Cukt 2.0, all of which are linked to artists, artist-researchers, art-tech collectives, or broader cultural experimentation ( Syntheticism.org 2025). In these cases, synthetic politics appears less as a straightforward attempt to build a durable electoral organisation and more as a form of political intervention conducted through aesthetic experimentation, performative critique, and symbolic disruption. The AI figure is often embedded in a wider creative project, and the surrounding organisational logic remains relatively open, fluid, and collective. This helps explain why these cases more often blur the line between politics, performance, and mediated public provocation.

A second cluster is civic-tech. Parker Politics, Lex AI, and AI Anno fit this group most clearly. Here the founders are more closely connected to civic technology, democratic innovation, software design, or participatory reform ( Syntheticism.org 2025). Compared with the artistic cluster, these projects are less oriented toward provocation and more toward practical experimentation with new forms of mediation between citizens and politics. What distinguishes this cluster is not simply technical competence, but the attempt to connect technical design with democratic reform. AI is therefore framed less as a symbolic intervention and more as a tool for improving representation, participation, and policy responsiveness.

A third cluster can be described as tech-oriented. This includes AI Mayor, AI Steve, and VIC. In these cases, the leading figures are more strongly associated with technology entrepreneurship, software development, or governance-oriented innovation. The political language is correspondingly more instrumental ( Schneier and Sanders 2025). These projects tend to frame AI as a means of improving political judgement, administrative capacity, informational processing, or decision-making consistency. Their founders’ backgrounds matter because they help explain why synthetic politics is presented here less as a critique of representation than as a practical response to the perceived inefficiencies and shortcomings of human government. Table 6 summarises the three founder-orientation clusters identified across the cases.

**Table 6.** Three clusters’taxonomy and founders’orientations.

Cluster	Founders’ orientation	Cases
Artistic	artists, artist-researchers, art-tech collectives, cultural experimenters	Leader Lars; Koneälypuolue; Olof Palme; Wiktoria Cukt 2.0
Civic-tech	civic-tech activists, democratic innovators, participatory tech designers	Parker Politics; Lex AI; AI Anno
Tech-oriented	technology entrepreneurs, software developers, governance-oriented innovators	AI Mayor; AI Steve; VIC

Source: Author’s own elaboration

### Organisational structure

The organisational structure of synthetic political actors remains generally thin and weakly institutionalised, even when some organisational elements extend beyond the AI interface itself. Across the ten cases, only two actors (AI Anno and AI Mayor) display signs of a more formalised organisational presence through an official office and registered party status. Even here, however, the degree of organisational depth differs substantially. AI Anno (e.g. Team Mirai) stands out as the most developed case in the sample, combining formal party status with an affiliated team, a visible social media ecosystem, a reported membership of 2,584, and a broader circle of 33,056 sympathisers (Team-mir.ai 2026). AI Mayor also shows a more formal organisational profile than most other cases, but on a much smaller scale, with only 30 members and no broader network of sympathisers ( Wikipedia contributiors 2026) Wikipedia.org.

By contrast, the remaining cases are better understood as thin political projects rather than as fully developed organisations. Most of them do not maintain an official office, registered membership, or a wider supporter structure. What exists instead is a looser organisational ecology built around affiliated teams and digital visibility. In seven of the ten cases, some form of affiliated team is identifiable, whether in the shape of artistic collectives, technical collaborators, or project-based support structures. These include groups such as Digital Democracy 2030, Computer Lars and MindFuture, The Center for Everything, Bombina Bombast, C.U.K.T., and NeuralVoice. Such teams suggest that synthetic political actors are rarely individual projects in a strict sense.

Social media ecosystems appear even more common. Eight out of ten cases show some kind of social media presence beyond the core AI interface. In these cases, visibility, circulation, and public contact are sustained through digital channels rather than through territorially embedded organisation. At the same time, the data also show the limits of this model. With the partial exception of AI Anno and, to a lesser extent, AI Mayor, synthetic political actors rarely convert digital visibility into formal membership or broader supporter bases. Organisationally, they therefore remain shallow: supported by teams and digital ecosystems, but only rarely institutionalised as durable political organisations. The main organisational features of the ten synthetic political actors are summarised in Table 7.

**Table 7.** Synthetic political actors‘main organisational features.

Case	Official office	Affiliated teams	Social media ecosystem	Membership	Sympathisers
AI Anno	Yes (registered party)	Digital Democracy 2030	Yes	2584	33056
AI Mayor	Yes (registered party)	No	Yes	30	No
AI Steve	No (non-registered party)	NeuralVoice	No	No	No
Leader Lars	No (non-registered party)	Computer Lars, MindFuture	Yes	No	No
Lex AI	No	No	Yes	No	No
Koneälypuolue	No	The Center for Everything	No	No	No
Olof Palme	No (non-registered party)	Bombina Bombast	Yes	No	No
Parker Politics	No	Parker Politics	Yes	No	No
VIC	No	No	Yes	No	No
Wiktoria Cukt 2.0	No	The collective C.U.K.T.	Yes	No	No

Source: Author’s own elaboration

### Internal participation

The participation dimension further illustrates how synthetic political actors depart from conventional political models. Citizens are most often invited to engage through chatbots, voicebots, social media interaction, or other forms of digital input. In this respect, participation is generally open and low-threshold, but also weakly institutionalised. Rather than acting as members in a stable organisation, participants appear more often as users who provide feedback, react to issues, or contribute to agenda formation through AI-mediated systems.

Three broad patterns can be identified. A first group consists of *interface-centred* cases, where participation remains almost entirely online and no meaningful offline participatory layer is present. AI Steve, Lex AI, and Parker Politics fall most clearly into this category. In these cases, participation is limited to mediated interaction through chatbots, voicebots, manifesto input, or social media engagement. A second group can be described as *electoral-institutional.* Here, online participation is combined with campaigning and electoral ambition, and in some cases participation is linked to a more conventional party trajectory. AI Mayor, VIC, and AI Anno fit this pattern, although AI Anno stands apart because its development culminated in Team Mirai as a more standard party organisation. These cases connect digital interaction with recognisable electoral politics, even if participation still remains more mediated and consultative than in conventional party organisations. A third group is *performative-artistic.* Leader Lars, Koneälypuolue, Wiktoria Cukt 2.0, and Olof Palme belong here. In these cases, participation extends beyond the interface through exhibitions, performances, pep rallies, and other public-facing artistic formats. Some of these projects may also intersect with electoral politics, but their participatory logic is shaped above all by public performance, symbolic intervention, and experimental engagement rather than by stable organisational incorporation.

Taken together, Table 8 suggests that synthetic political actors do not simply digitise older forms of internal party participation. They reorganise participation more fundamentally. What matters is less membership or organisational embeddedness than the capacity to attract interaction, collect input, and channel it through technological systems. Even where participation becomes more elaborate, it rarely develops into a durable organisational relationship. The dominant pattern therefore remains clear: participation is primarily online, mediated, and weakly institutionalised, while offline participation appears either in electoral-institutional form or through performative and artistic public engagement.

**Table 8.** Internal participation of synthetic political actors.

Cluster	Case	Online participation	Offline participation
**Interface-centered **	AI Steve	Manifesto creation; voicebot interaction	None
	Lex AI	Chatbot interaction; social media interaction	None
	Parker Politics	Chatbot interaction; social media interaction	None
**Campaign-linked **	AI Anno	Chatbot and voicebot interaction; gamification activities; manifesto creation; social media interaction	Campaigning; electoral candidacy; membership
	AI Mayor	Chatbot interaction; social media interaction	Campaigning; electoral candidacy; membership
	VIC	Chatbot interaction; social media interaction	Campaigning
**Performative hybrid**	Leader Lars	Chatbot interaction; social media interaction	Art exhibitions and performances; campaigning; electoral candidacy
	Koneälypuolue	Chatbot interaction	Art exhibitions and performances
	Olof Palme	Voicebot interaction	Campaigning; electoral candidacy; pep rallies
	Wiktoria Cukt 2.0	Voicebot interaction; social media interaction	Art exhibitions and performances

Source: Author’s own elaboration

## Discussion

The empirical analysis of ten synthetic political candidates and parties across multiple national contexts generated several findings that together advance our understanding of emerging synthetic political actors in contemporary democracies. This discussion reflects on four interrelated themes, as mentioned in the introduction: the internal diversity and structural logic of synthetic politics; the contribution to theoretical debates on AI and democratic delegation; the broader implications for disintermediation dynamics; and what these findings suggest about the accelerating weakening of political parties as linkage institutions.

First, the comparative mapping reveals that, so far, synthetic political actors are neither a coherent ideological family nor a uniform organisational type. They range from durable electoral organisations such as Team Mirai, to more symbolic and performative interventions that use the appearance of candidacy without credible office-seeking ambitions. The typology of political agency developed here and refined empirically, distinguishing autonomous synthetic actors, digital replicas, and hybrid human-AI formations, moves beyond the binary of “AI as tool versus AI as actor” that has characterised much of the prior literature ( García-Marzá and Calvo 2024, Vuković 2025). Similarly, our three-cluster taxonomy of normative assumptions (participatory-representational, technocratic-governance, and art-political/post-human) captures a dimension that existing studies have either neglected or treated as a residual category. In doing so, this paper offers the first structured, multi-dimensional comparative assessment of synthetic political actors, constituting both a theoretical and methodological step forward from predominantly single-case or two-cases comparative research that has dominated the field.

Second, our findings speak directly to the theory of AI and delegation in democracy. Delegation theory, in its classical formulation, assumes a principal-agent chain running from citizens to elected representatives, whose accountability is institutionally secured through regular and fair elections ( Grossman & Hart 1983, Strøm 2000). Synthetic political actors disrupt this chain in at least two ways. The first is what this study terms configurational ambiguity: it is genuinely unclear who is delegating to whom when a citizen interacts with an autonomous AI interface presented as a candidate. The human designers retain de facto control over the system’s parameters, yet they are organisationally invisible. The citizen-facing AI persona performs political agency without being legally accountable. This tripartite arrangement, between citizen, synthetic interface, human backstage operator, creates a new layer in the delegation chain that existing democratic theory does not adequately account for.

The second disruption concerns representation itself. Several cases examined here, and notably Leader Lars, VIC, and Wiktoria Cukt 2.0, either explicitly reject or radically reframe the notion that representation requires a human intermediary. Participation is reconstituted as data input, preference aggregation through algorithmic mediation, or proxy representation, transforming citizens from principals who delegate to inputs within a computational system. This finding has direct implications for normative democratic theory and for regulatory frameworks that still assume human candidacy as the default unit of electoral accountability ( Altman 2026).

Third, the pattern of weak institutionalisation and interface-centric organisation documented across most cases connects directly to wider disintermediation dynamics in contemporary politics ( Gerbaudo 2018 and 2024). The overwhelming majority of synthetic political actors maintain no formal membership, no territorial branches, and no stable organisational infrastructure beyond affiliated teams and social media ecosystems. What generates political presence is not organisational depth but interface reach: the ability of the AI system to make itself available, responsive, and addressable at scale. This constitutes a qualitatively distinct form of political presence, which now no longer requires the complex horizontal networks that party organisation traditionally provided. Disintermediation, understood here as the progressive bypassing of institutional intermediaries in the relationship between citizens and political representation ( Barberà et al. 2021, Deseriis 2020), does not simply describe a technological affordance. It is a structural feature of synthetic political actors whose very architecture assumes that the human-to-human organisational layer of democratic politics is either inefficient, biased, or simply unnecessary. The evidence across all three entrepreneurial clusters, artistic, civic-tech, and tech-oriented, converges on this point, even where the explicit normative framing differs considerably.

Finally, and perhaps more importantly, the findings show the progressive weakening of political parties as linkage institutions. The classic linkage function, connecting citizens to the state through organised aggregation of interests, socialisation, mobilisation, and representation ( Lawson 1980, Scarrow et al. 2017), assumes that parties are the primary site at which diffuse political preferences are translated into governable programmes and electoral mandates. Synthetic political actors do not simply compete with parties for votes; they contest the very premise of party-mediated linkage. By positioning AI interfaces as the locus of preference aggregation, agenda formation, and political representation, they propose a model in which the organisational infrastructure of the party, its membership, its leadership, its ideological coherence, is replaced by algorithmic mediation. Even in the most institutionally ambitious case, Team Mirai, the synthetic dimension is not incidental to the organisation, but constitutive of its claim to represent. The cases analysed here thus suggest that the weakening of party linkage is not merely a story of declining membership or partisan dealignment; it is being actively accelerated by the emergence of new political actors whose organisational logic explicitly positions the party form as an obstacle to genuine representation rather than its main instrument.

## Conclusion

This article has provided the first systematic comparative analysis of synthetic political actors, AI-based candidates and parties that present algorithmic interfaces as the primary locus of political agency, representation, and citizen engagement. Drawing on ten cases across eight national contexts and six analytical dimensions, it has demonstrated that these initiatives are neither a uniform phenomenon nor a marginal curiosity of the political fringe. They constitute a distinct, growing and rapidly diversifying category of political actor that challenges foundational assumptions of democratic theory, party organisation, and electoral representation.

The theoretical contribution is threefold. First, the analytical framework developed here, covering political agency, normative assumptions, entrepreneurship, organisational structure, internal participation, and political goals, provides a replicable comparative toolkit that goes substantially beyond existing case-specific accounts. Second, the empirical analysis reveals that synthetic political actors reconfigure the classical principal-agent chain of democratic delegation by introducing an algorithmically mediated layer that is publicly visible, but organisationally unaccountable in conventional terms. Third, this study situates synthetic political actors within the longer arc of democratic disintermediation: the progressive displacement of party-mediated linkage by interfacial, data-driven, and algorithmically aggregated forms of political engagement. The evidence presented here suggests that this displacement is no longer hypothetical or experimental.

These findings carry significant implications for both democratic theory and regulatory practice. Theoretically, they demand an extension of delegation and linkage frameworks to account for non-human agents as politically addressable actors. Normatively, they raise urgent questions about accountability, transparency, and the conditions under which AI-mediated representation can be considered democratically legitimate. Regulatorily, existing electoral law in most democracies continues to presuppose human candidacy as the unit of accountability; the emergence of synthetic political actors exposes this as an increasingly inadequate assumption. Future research should extend the comparative scope of this study, track the institutional trajectories of surviving cases, and engage more directly with the legal and regulatory architectures (or their absence) that condition how synthetic political actors enter and persist in democratic electoral system.

## Ethics and consent

Ethical approval and consent were not required for this study. The study is based primarily on qualitative document analysis of publicly available materials.

## Data Availability

The data underlying the results of this study are available on Zenodo: Synthetic candidates and political parties. https://doi.org/10.5281/zenodo.19521950. Malý, M. (2026). The dataset contains the comparative case-level evidence used in the analysis of the ten synthetic political actors examined in this article. Data are available under the terms of the Creative Commons Attribution 4.0 International licence (CC BY 4.0).
