# Supplementary material for: Synthetic political actors: an exploration into emerging AI-driven candidates and parties
Source: Open Res Eur. 2026 Apr 30;6:138. [Version 1] doi: 10.12688/openreseurope.23686.1 (PMC13191310; doi:10.12688/openreseurope.23686.1)
Supplement: Peer review history

# Reviewer report: Reviewer response for version 1

**DOI:** 10.21956/openreseurope.25644.r73537

**Published:** 2026-05-25

**Authors:** Ben O'Loughlin

**Affiliations:** 1 Royal Holloway University of London, Egham, UK

This is a valuable paper precisely in the terms it self-describes: this is an exploratory paper trying to map the basic features of synthetic political actors. This can stimulate more research in this field because through comparative analysis across countries and types of synthetic political actors because it illuminates something we know little about and this can push researchers to explore this in more detail. I am also pleased the authors give attention to how we theorize and understand representation, authority and other phenomena in the context of how we define democracy. 

 My only suggestion for revision is that on p6 the authors talk about six dimensions, on p7 six analytical dimensions, but earlier on p5-6 we were given four features, labelled a, b, c and d. Are the other two features earlier in the text, and if so, why were they not given and e and f? I read the first half as giving two features and four dimensions. If others are to use this as an analytical framework this needs to be clarified. 

 I was going to recommend putting links of each political actor to its website, but we can ctrl+f search and find them, so no need. 

 I hope this is indexed soon so I can share it with researchers in this field.

If applicable, is the statistical analysis and its interpretation appropriate?

Not applicable

Is the study design appropriate and is the work technically sound?

Yes

Is the work clearly and accurately presented and does it engage with the current literature?

Yes

Are the conclusions drawn adequately supported by the results?

Yes

Are sufficient details of methods and analysis provided to allow replication by others?

Yes

Are all the source data and materials underlying the results available?

No source data required

Reviewer Expertise:

I edit a book series AI Politics for Intellect Books / University of Chicago Press.

I confirm that I have read this submission and believe that I have an appropriate level of expertise to confirm that it is of an acceptable scientific standard.

# Reviewer report: Reviewer response for version 1

**DOI:** 10.21956/openreseurope.25644.r73540

**Published:** 2026-05-20

**Authors:** Silvija Vuković

**Affiliations:** 1 University of Zagreb, Zagreb, Croatia

This article is well written: timely, relevant, engaging, and analytical. It offers a strong categorization and conceptualization of emerging AI-driven candidates. The theoretical section is well informed, clearly presented, and grounded in the relevant literature. The methodology and case selection are appropriate.

 My only suggestion is to carefully proofread the manuscript once more, as there are occasional sentences that appear unfinished or unclear. For example, the following two cases could be revised for clarity:

 "The aim of this article is to addresses this gap by engaging into a more in-depth theoretical exploration and empirical assessment of the nature and key features of, ten synthetic political candidates and parties across multiple national contexts."

 "In this regard, the literature has so far identified as synthetic political actors all those political initiatives organised around AI-based synthetic interfaces that are presented as recognisable political personae and serve as central points of political engagement constitute what the literature ( Staunæs and Herrie 2024, Vuković 2025)."

 I highly suggest indexing this article.

If applicable, is the statistical analysis and its interpretation appropriate?

Not applicable

Is the study design appropriate and is the work technically sound?

Yes

Is the work clearly and accurately presented and does it engage with the current literature?

Yes

Are the conclusions drawn adequately supported by the results?

Yes

Are sufficient details of methods and analysis provided to allow replication by others?

Yes

Are all the source data and materials underlying the results available?

Yes

Reviewer Expertise:

Political communication, media studies

I confirm that I have read this submission and believe that I have an appropriate level of expertise to confirm that it is of an acceptable scientific standard.

# Reviewer report: Reviewer response for version 1

**DOI:** 10.21956/openreseurope.25644.r73536

**Published:** 2026-05-20

**Authors:** Stine Ziegler

**Affiliations:** 1 Philipps University Marburg Department of Political Science (Ringgold ID: 153764), Marburg, Hesse, Germany

This article addresses the very innovative and timely topic of how AI models are being staged as an enhancement of even a replacement of political actors. By providing a systematic analysis of ten so-called *synthetic candidates* across nine countries, it offers valuable empirical insight into a novel mode of AI-driven political influence in democracies. The study lays important groundwork for the assessment of future developments of AI being used as an interface between citizens and democratic institutions. As such, the article constitutes a relevant and important contribution to research on new forms of democratic linkage in the age of AI.

 Nevertheless, three aspects could further strengthen the overall argument and its contribution to the literature:

1. Given that *synthetic candidates* represent a very recent phenomenon, the article would benefit from a clearer and more detailed explanation of the concept at an earlier stage. I recommend including two or three illustrative examples representing the spectrum of AI-driven candidates in the introduction. This would help readers’ understanding of what you are writing about before turning to the analysis, where the concept already becomes much clearer.
2. Synthetic candidates raise fundamental questions about democratic representation and legitimacy. Therefore, the article would benefit from a more explicit and systematic engagement with democratic theory. I recommend focussing more on the relation between human and synthetic candidates, questions on accountability and participatory rights. What problems of democracies or, more precisely, of political parties do *synthetic candidates* solve? What democratic norms are incorporated into the AI models? What potential unintended consequences or democratic risks might arise from their use. These issues are already present in the paper, but they remain somewhat implicit. Bringing them to the forefront and framing them as a core theoretical contribution would significantly strengthen the argument. The article of Dommett and Rye (2018) (Ref 1) could be an inspiration in this regard.
3. Be careful not to humanise AI. Throughout the paper I noticed several statements that are phrased as if synthetic candidates had any kind of agency or consciousness. While this may partly be a matter of writing style, these kinds of statements blur the effect of politicians/developers/organisations making choices, training the models and hence being accountable for how the AI candidates work. A more precise conceptualisation of AI as a socio-technical system embedded in organisational and political contexts would allow for a more nuanced analysis of evolving power dynamics and their democratic implications.
  For example,
  - “[…], the paper examines the main goals of this emerging group of political actors, how they articulate political representation, how political agency is distributed between AI systems and human actors, or how they conceive internal organisation and participation.” (p. 3) – AI systems do not articulate representation or conceive participation. These are decisions made by human actors (politicians or developers) and implemented in the design and training of the models.
  - “[…] this dimension examines what kind of political aims synthetic political actors pursue.” (p. 5) – It is not the synthetic actors themselves that pursue political aims, but the organisations behind them.

If applicable, is the statistical analysis and its interpretation appropriate?

Not applicable

Is the study design appropriate and is the work technically sound?

Yes

Is the work clearly and accurately presented and does it engage with the current literature?

Partly

Are the conclusions drawn adequately supported by the results?

Partly

Are sufficient details of methods and analysis provided to allow replication by others?

Yes

Are all the source data and materials underlying the results available?

Yes

Reviewer Expertise:

political parties, participation, digital democracy

I confirm that I have read this submission and believe that I have an appropriate level of expertise to confirm that it is of an acceptable scientific standard, however I have significant reservations, as outlined above.

## References

[1] : Taking up the baton? New campaigning organisations and the enactment of representative functions. *Politics* .2018;38(4) : 10.1177/0263395717725934 411-427 10.1177/0263395717725934

# Reviewer report: Reviewer response for version 1

**DOI:** 10.21956/openreseurope.25644.r73534

**Published:** 2026-05-20

**Authors:** Francesco Nasi

**Affiliations:** 1 University of Bologna, Bologna, Italy

I thank the authors for work they have conducted for this article, which I found highly original, well written, clear, and particularly timely in the current debate on AI systems and democracy. The paper systematizes and provides a mapping of existing synthetic political actors. Drawing on the literature on party politics, it develops a clear analytical framework encompassing political agency, normative assumptions, entrepreneurship, organizational structure, internal participation, and political goals. One of the article’s major strengths lies in its positioning within the tradition of party politics scholarship, together with an overall engagement with scholarship on AI and democracy. This allows the authors to situate a new phenomenon within an established conceptual framework, thereby making it more analytically intelligible and theoretically grounded.

 I would only like to offer a few minor observations that would probably go under the “minor revision” label.

 1) The selection criteria appear appropriate and well justified. In particular, distinguishing cases in which actors have actually run for office (or have explicitly declared the intention or objective to do so) helps exclude initiatives that remain primarily experimental in nature. I am thinking, for instance, of cases such as the AI Mayor of Venice ( https://sindaco-ai.vercel.app/) or the AI Mayor of Taranto ( https://www.instagram.com/annalucedamico/).  There may, however, be room for slight refinement of the first criterion, namely: “the initiative presents an AI-based persona as a political candidate or party actor (e.g. party leader).”

 One potential limitation of this criterion in its current formulation is that it may exclude cases that could fall within the broader category of synthetic politics. For example, Andrew Gray ( https://www.politico.eu/article/britain-uk-andrew-gray-ai-artificial-intelligence-manifesto-polis-mp-nigel-adams-selby-ainsty-election/) does not fully satisfy the first criterion in the strict sense of presenting an “AI persona.” However, he is a human political actor who explicitly stated his intention to operate in this manner, delegating a substantial part of his political agency to the evaluations produced by the Pol.is system based on citizens’ inputs and preferences. In this way, he developed an electoral manifesto for his candidacy in the UK constituency of Selby and Ainsty in 2023. A similar example is Team Mirai in Japan, which systematically integrates AI into its decision-making processes while also maintaining elected human representatives, and which has been included in the mapping.

 Can these cases still be considered examples of synthetic politics even when the AI system is not itself the primary political actor? In order to accommodate cases such as Andrew Gray’s candidacy and Team Mirai more adequately, the first criterion could perhaps be expanded as follows (or something like this): “the initiative presents an AI-based persona as a political candidate or party actor (e.g. party leader), or uses AI tools as a fundamental element of its decision-making and agenda-setting processes.”

 2) Compared to the other cases examined, AI Anno and Team Mirai more generally appears to operate at a more advanced level, with 11 elected parliamentary representatives, a formally established party structure, statutes, and organizational mechanisms. I believe this qualitative difference deserves to be acknowledged more explicitly, perhaps in the “Discussion” section of the paper.

 3) Another point on which I would encourage the authors to reflect further concerns the extent to which the three clusters of normative assumptions are mutually exclusive, or whether they should instead be understood as partially overlapping. The authors correctly observe that: “the ten cases do not point to a single normative model of synthetic politics. They fall more convincingly into three clusters that differ in how they justify the political role of AI.”

 These three clusters are identified as: 1) broadening representation; 2) supporting technocratic governance; and 3) questioning the human foundations of politics.

 It may, however, be more accurate to suggest that these clusters reflect different emphases placed on particular dimensions, rather than entirely distinct normative categories. Most of the cases likely share at least some of these underlying elements: for instance, a technocratic rationale, or a desire to renew and reconfigure democratic participation. Wiktoria Cukt 2.0 and the Finnish AI party seems to me the only cases that adopts a more explicit anti-participatory stance. However, they clearly sharing a technocratic rationale. By contrast, the Olof Palme Project (which is placed within the same cluster as Wiktoria and the Finnish AI Party) explicitly invites greater citizen participation. As stated in the cited source: “ *We want to create personal meetings between visitors and our virtual party leader to suggest what democratic open conversation could be in an age of artificial intelligence and new digital publics. We need to hear what an experienced reformist socialist would say in an election campaign that, no matter what we do, will be orchestrated by how the vast amounts of data we provide are mirrored and distorted. This is what Emma Bexell, representative of the AI Party and artist known for her cross-border performing arts, says. The AI Party's new leader adds: ‘Democracy must therefore be constantly won anew.*’”

 I do not see a need to revise the categories themselves, which appear convincing at this level of analysis. Rather, I would suggest framing them differently, not as mutually exclusive, but as configurations characterized by different normative emphases, while also recognizing that some of these assumptions may at times come into tension with one another, as appears to be the case with Wiktoria Cukt 2.0 and the Finnish AI Party.

 4) A further issue that I believe would be very interesting to explore (perhaps briefly in the conclusion, as a possible avenue for future research) concerns the extent to which these initiatives are actually capable of achieving political success and institutional relevance. For instance, why is the case of AI Anno so markedly different from the others? Is this primarily related to the specific cultural context of Japan and its political system, or does it depend more on Anno’s own role as a political entrepreneur? Building on the mapping developed in this article, it would be valuable in the future to pursue more in-depth qualitative analyses of individual cases in order to identify their underlying conditions of possibility. Such an approach could also help clarify the extent to which synthetic political actors may realistically become politically relevant actors in the future, or whether their impact is likely to remain marginal.

 5) As noted previously, the analytical dimensions are well justified and articulated. I would only add a minor stylistic observation. In the section “Assessing the nature and main features of emerging synthetic political actors”, the first two dimensions (political agency and normative assumptions) are not labeled with letters, whereas the subsequent dimensions are marked alphabetically (a, b, c, d). It may be preferable either to label all dimensions consistently or not to use lettered labels at all, in order to avoid potential confusion or ambiguity.

If applicable, is the statistical analysis and its interpretation appropriate?

Not applicable

Is the study design appropriate and is the work technically sound?

Yes

Is the work clearly and accurately presented and does it engage with the current literature?

Yes

Are the conclusions drawn adequately supported by the results?

Yes

Are sufficient details of methods and analysis provided to allow replication by others?

Yes

Are all the source data and materials underlying the results available?

Yes

Reviewer Expertise:

Sociology of AI, democratic AI, democratic innovations, social imaginaries

I confirm that I have read this submission and believe that I have an appropriate level of expertise to confirm that it is of an acceptable scientific standard.
